# An audit of medullary thyroid carcinoma from a tertiary care hospital in northwest India

**DOI:** 10.3389/fendo.2023.1226348

**Published:** 2024-01-08

**Authors:** Ananda Mohan Chakraborty, Ashutosh Rai, Rimesh Pal, Soham Mukherjee, Divya Dahiya, Rajinder Kumar, Uma Nahar Saikia, Naresh Kumar Panda, Sanjay Kumar Bhadada, Pinaki Dutta

**Affiliations:** ^1^ Department of Endocrinology, Postgraduate Institute of Medical Education and Research, Chandigarh, India; ^2^ Centre for Endocrinology, William Harvey Research Institute, Queen Marry University of London, London, United Kingdom; ^3^ Department of General Surgery, Postgraduate Institute of Medical Education and Research, Chandigarh, India; ^4^ Department of Nuclear Medicine, Postgraduate Institute of Medical Education and Research, Chandigarh, India; ^5^ Department of Histopathology, Postgraduate Institute of Medical Education and Research, Chandigarh, India; ^6^ Department of Otorhinolaryngology, Postgraduate Institute of Medical Education and Research, Chandigarh, India

**Keywords:** medullary thyroid carcinoma, neuroendocrine tumor, tyrosine kinase inhibitor, calcitonin, *RET* mutation

## Abstract

**Introduction:**

Medullary thyroid carcinoma (MTC) is a rare thyroid malignancy originating from parafollicular C cells. It accounts for 5%–10% of all thyroid malignancies.

**Methods:**

An ambispective analysis of pathologically proven MTC presented in a tertiary care hospital in northwest India was performed after considering demography, clinical manifestation, *RET* mutation status, management, and outcome as denominators.

**Results:**

Among 2,735 thyroid malignancy cases who presented to our institute in the last 10 years (2012–2022), 78 (3%) had MTC with a mean age of presentation of 43 ± 11 years; 60% of them were female. The median duration of symptoms was 23 months (IQR 12–36 months). The most common presenting complaint was goiter with lymphadenopathy (80.8%). Among the atypical presentations, one each had ectopic Cushing’s syndrome, hypertensive crisis in pregnancy due to pheochromocytoma, synchronous chondrosarcoma, and Von Hippel–Lindau disease spectrum. Median calcitonin and carcinoembryonic antigen (CEA) levels at presentation were 1,274 pg/mL (*n* = 64) and 149 ng/mL (*n* = 39), respectively. Twenty-two patients were germline *RET* mutation-positive, and they presented at a younger age. Majority of the patients presented with stage IV disease. Surgery was the primary modality of therapy. Twenty-nine patients received radiotherapy and 25 patients received tyrosine kinase inhibitors (TKIs). Nine patients received peptide receptor radiotherapy (PRRT) with Lu-177 with neoadjuvant capecitabine. Median progression-free survival (PFS) was 60 months. Patients without structurally and biochemically residual disease and stable disease after the first modality of therapy (Log-rank 11.4; *p* = 0.004) had a better PFS. Female patients (Log-rank: 9.5; *p* = 0.002) had a better PFS than male patients.

**Conclusion:**

This study showed that MTC comprises 3% of thyroid malignancies with a female preponderance. *RET* mutation-positive patients had a younger age at presentation. Surgery was the first-line therapy. Radiotherapy, TKI, and PRRT were given as a part of second-line or third-line therapy due to persistent disease and/or disease recurrence. The median PFS was better in female patients and in patients who had no residual lesions and stable disease after the primary modality of therapy.

## Introduction

Thyroid cancer has already become the 10th most common cancer worldwide. The incidence rate of thyroid cancer in 2020 was 10.1 per 100,000 women and 3.1 per 100,000 men. Ninety percent of thyroid cancers are differentiated thyroid carcinoma (1, 2). Though medullary thyroid carcinoma (MTC) comprises 5% to 10% of thyroid malignancies (3, 4), it accounts for 15% of thyroid cancer-related mortality (3, 4). Nearly 80% of MTCs are sporadic; the remaining 20% are inherited as a multiple endocrine neoplasia (MEN) spectrum. The most common presentation is neck swelling often with an indolent course. Since goiter is very common in various parts of the world, diagnosis of MTC is often delayed until it reaches an advanced stage. Even if it is diagnosed early, it is usually reported as suspicious of malignancy or cellular atypia by fine needle aspiration, unless a calcitonin staining becomes positive or demonstrable amyloid stroma is present. Therefore, diagnostic delay with poor therapeutic outcome is not infrequent.

Total thyroidectomy is the only effective therapy for MTC. The cure depends on the promptness of diagnosis, limited lymph node involvement, and radical surgical treatment. There has been a paradigm shift in the management of MTC with the advent of tyrosine kinase inhibitors (TKIs), *RET*-specific inhibitors, and peptide receptor-based radiotherapy. Surgery and conventional radiotherapy are still considered the standard therapy in countries with limited resources like India.

Experience of MTC management is varied among different parts of the world; even a regional difference has also been described in the USA and in Europe. Since India is a country with a heterogeneous population, a difference in the presentation, management, and outcome of MTC in different parts of India is expected.

This is an ambispective analysis with the objective to describe the demography, clinical manifestation, *RET* mutation status, management, and outcome of pathologically confirmed cases of MTC patients attending a tertiary care hospital in northwest India.

## Methodology

The medical records and clinical data of pathologically proven cases of MTC patients registered in the Postgraduate Institute of Medical Education and Research, Chandigarh between 2012 and 2022 were analyzed. Written informed consent was taken from patients and from their relatives in case of demise. The study was approved by the institutional ethical committee (GOET: IEC/O7/2020/1715).

All histopathologically and cytopathologically proven cases of MTC that were operated in this institute or referred from outside and subsequently managed in this hospital were included in this study. For patients who were operated outside, a histopathological confirmation was done by a designated histopathologist (UNS).

Demography, clinical presentation, radiology, histopathology, and cytopathology data at the time of presentation were analyzed. Serum calcitonin and CEA level were determined preoperatively and 3–6 months postoperatively. All such available results were analyzed and repeated at the time of their last presentation whenever feasible. Calcitonin and CEA were analyzed by ECLIA (ELECSYS-2010, Roche, Germany Cobas e 411). Any value above the upper limit of the normal laboratory reference limit was considered elevated in an appropriate setup.

Tumor staging was done based on tumor–node–metastasis (TNM) classification (5). Tumor volume was calculated in patients having single lesions on imaging with the following formula: Antero-posterior diameter × Transverse diameter × Supero-inferior diameter × 0.523.

Genetics for *RET* mutation status was done in patients with positive family history, syndromic patients, and patients with clinical suspicion.

Treatment modalities and outcome data of available cases were also explored.

Disease remission was considered when the serum calcitonin and CEA levels were lower than the normal laboratory limit and there was no residue in contrast-enhanced computer tomography (CECT) from the base of the skull to the pelvis and/or 68 Ga DOTA positron emission tomography-computer tomography (68 Ga DOTA PET-CT) not showing any tracer avid lesion suggestive of metastatic disease. Stable disease and progressive disease were considered as per RECIST 1.1 (5). The first follow-up after the primary modality of therapy was usually done at 3 months and subsequent follow-up was done at 3- to 6-month intervals.

The biochemically persistent disease was considered if calcitonin and CEA were elevated above the laboratory reference range and there was no residue either on CECT or on 68 Ga DOTA PET-CT. Isolated CEA elevation was considered as a de-differentiation of the tumor and all such cases were confirmed either by CECT or by 68 Ga DOTA PET-CT.

At the time of the last follow-up, patients were categorized as having remission, progressive disease, stable disease, succumbed to MTC, succumbed to other causes, and not available for follow-up. For progressive or stable but persistent disease, biochemical and structural persistence were analyzed separately.

### Statistics

All analyses were conducted by using the Statistical Package for Social Sciences (version 21.0). The normality of data was assessed by the Shapiro–Wilk test. Upon determination of normality, descriptive statistics in the form of mean or median were presented accordingly. A comparison of two continuous variables was done by the Student’s *t*-test and Mann–Whitney *U*-test for normally and non-normally distributed data, respectively. For the categorical variable, a chi-square test was performed. Survival analysis was done by the Kaplan–Meier method. *p* < 0.05 was considered statistically significant.

## Results

Among 2,735 patients with thyroid malignancy, 78 (3%) pathologically proven MTC patients were presented to this hospital from 2012 to 2022. Forty-seven of those were female (60%). The mean age of the patients at the time of presentation was 43 ± 11 years (range, 19–71 years). The mean age at presentation for male and female patients was 46 years and 42 years, respectively. Median duration of symptoms at the time of presentation was 23 months (IQR 12–36 months). Family history of malignancy was present in eight patients: five had a positive family history of thyroid malignancy, and the other three patients had a family history of breast carcinoma, colon carcinoma, and skeletal malignancy.

The most common presenting complaint was goiter with lymphadenopathy in 63 (80.8%) patients; weight loss and diarrhea were the presenting complaints in four and two patients respectively. Two asymptomatic patients with a family history of MEN were diagnosed as a part of the family screening. Among the unusual presentation, one patient presented with ectopic Cushing’s syndrome, two patients had thyrotoxicosis, one had hypertensive crisis presenting during pregnancy, one had prostatic carcinoma, and MTC was detected incidentally during a prostate-specific membrane antigen (PSMA)-PET scan.

Apart from these presenting complaints, some of the MTC patients had associated malignancies, for example, clear-cell type of renal cell carcinoma, and chondrosarcoma and intraductal breast carcinoma were seen in one patient each (**Table 1**).

**Table 1:** Distribution of MTC patients as per their associated thyroid disease, HPE findings of thyroid lesions, and other organ diseases.

Cases	Stage of MTC at diagnosis	Other HPE findings (from neck lesion)	Other organ diseases	Associated thyroid disease
54/Female	III	NA	NA	Graves’ disease
54/Male	IV C	PTC	NA	PTC
48/Female	II	Follicular adenoma	NA	Follicular adenoma
39/Male	NA	NA	Angiomyolipoma	NA
39/Female	III	NA	NA	Hyperthyroidism
66/Female	NA	Follicular neoplasm	NA	Follicular neoplasm
40/Female	NA	Lymph node tuberculosis	Lymph node tuberculosis	NA
55/Female	NA	NA	NA	Hyperthyroidism
61/Male	IV C	NA	Ectopic Cushing’s	NA
24/Female	IV A	NA	Hypertension in pregnancy and hypertensive encephalopathy	NA
34/Female	IV A	PTC	Infiltrating ductal carcinoma of breast	PTC
59/Female	III	PTC	NA	PTC
63/Male	IV A	NA	Carcinoma prostate	NA
42/Female	IV B	NA	RCC (B/L), pancreatic cyst, VHL mutation	NA
48/Male	IV A	NA	Chondrosarcoma	NA

PTC, papillary carcinoma of thyroid; RCC, renal cell carcinoma; NA, not available/applicable; VHL, Von Hippel–Lindau; HPE, histopathological examination.

Majority of them were diagnosed on histopathology, fine needle aspiration cytology (FNAC) was done in 38 patients who presented with solitary thyroid nodule, and ultrasonography was suspicious of malignancy. Twenty-two (58%) of them were diagnosed to have MTC on cytopathology; four had a diagnosis of differentiated thyroid carcinoma. FNAC reported five patients as having a benign disease, but due to strong clinical suspicion of malignancy and elevated calcitonin and/or CEA, they were subjected to surgery and later found to have MTC on histopathology.

Among available data (*n* = 72), 29 (40.3%) patients had right lobe involvement, and 27 (37.4%) and 16 (22.2%) had left lobe and both lobe involvement, respectively, as per post-surgical histopathological assessment. The median tumor volume (*n* = 24) was 9.8 cc (IQR 5.5–46.6 cc). The median serum calcitonin level was 1,274 pg/mL (IQR 358–4,104 pg/mL) (*n* = 64) with the highest value encountered being 42,697 pg/mL and the median CEA level was 149 ng/mL (IQR 30–316) (*n* = 39).

Germline *RET* protooncogene mutation assessment was done for 47 patients, 22 of whom had the mutation-positive disease. One patient had Erβ mutation positivity. Twelve patients had MEN 2A and three had MEN 2B in this cohort; the remaining seven cases were initially considered a sporadic disease, and later found to have germline *RET* mutation positive. The most common *RET* mutation detected was P.C634Y followed by P.V804M.

The mean age at presentation for *RET* mutation-positive cases was 37 ± 12 years. No meaningful relationship existed between the duration of symptoms, pre-operative calcitonin, and CEA level with *RET* mutation status in this cohort (**Table 2**).

**Table 2:** Distribution of study subjects according to their *RET* mutation status (*N* = 47).

Attributes	*RET* mutation negative (*n* = 25)	*RET* mutation positive (*n* = 22)	Statistics
Age	45 ± 11	37 ± 12	*p* = 0.027^a^
Pre op calcitonin, pg/mL (median)	1,375 (IQR 7,786)	1,051 (IQR 1,366)	*p* > 0.05^b^
Pre op CEA, ng/mL (median)	34 (IQR 139)	149 (IQR 266)	*p* > 0.05^b^
Duration of symptoms, months (median)	12 (IQR 43)	23 (IQR 66)	*p* > 0.05^b^

a t-test.

b Mann–Whiney U test.

Among the germline *RET* mutation-positive patients, seven patients had cutaneous lichen amyloidosis, six each had pheochromocytoma, cutaneous lichen amyloidosis, and hyperparathyroidism. Two patients had mucosal neuroma (**Table 3**).

**Table 3:** Distribution of *RET* mutation-positive cases in this cohort as per their demographic, tumor stage, MEN component, and mutation (*n* = 15).

Age/Gender	Tumor STAGE	MEN component
26/Female	IV C	MTC, CLA
49/Female	III	MTC, PHPT
30/Female	IV A	MTC, Pheochromocytoma, PHPT, CLA
35/Male	IV C	MTC, Pheochromocytoma, Mucosal neuroma, Megacolon
40/Female	NA	MTC, PHPT, CLA
24/Female	IV A	MTC, Pheochromocytoma, CLA
31/Male	II	MTC, Pheochromocytoma, Mucosal neuroma
24/Female	NA	MTC, Pheochromocytoma
24/Female	III	MTC, CLA
36/Female	III	MTC, Pheochromocytoma, PHPT
27/Female	III	MTC, CLA
36/Male	IVC	MTC, CLA, PHPT
42/Female	IVB	MTC, PHPT
29/Female*	NA	MTC
39/Male*	NA	MTC

MTC, medullary thyroid carcinoma; CLA, cutaneous lichen amyloidosis; PHPT, primary hyperparathyroidism; NA, not available.

Seven patients were suspected to have sporadic MTC without other clinical markers turning out to be RET mutation positive.

*Both the patients presented with medullary thyroid carcinoma as sole manifestation of MEN and family history positive for MTC had mutation in Codon 634 of Chromosome 10q11.2.

Tumor staging at presentation was available for 71 patients; 26 (33%) of them had stage 3 disease, 18 (23%) had stage 4A disease, and 14 (18%) and 9 (11.5%) patients had stage 4B and 4C disease, respectively. The most common site of metastasis was locoregional lymph nodes (66%) followed by vertebrae (18%), lung (9%), and liver metastasis (5%). Male patients (27/30) had more stage IV disease as compared to female patients (20 out of 41) (chi-square: 13.14; *p* < 0.001).

^68^Ga DOTA positron emission tomography (DOTA-PET) was started in 2012 in this institute. Since then, all potential neuroendocrine tumor patients have undergone DOTA-PET for staging and consideration of further therapy; 59 patients underwent DOTA-PET in this study for tumor localization and for assessment of metastatic disease (some patients who underwent primary surgery outside our hospital did not undergo DOTA-PET).

**Figure 1:**
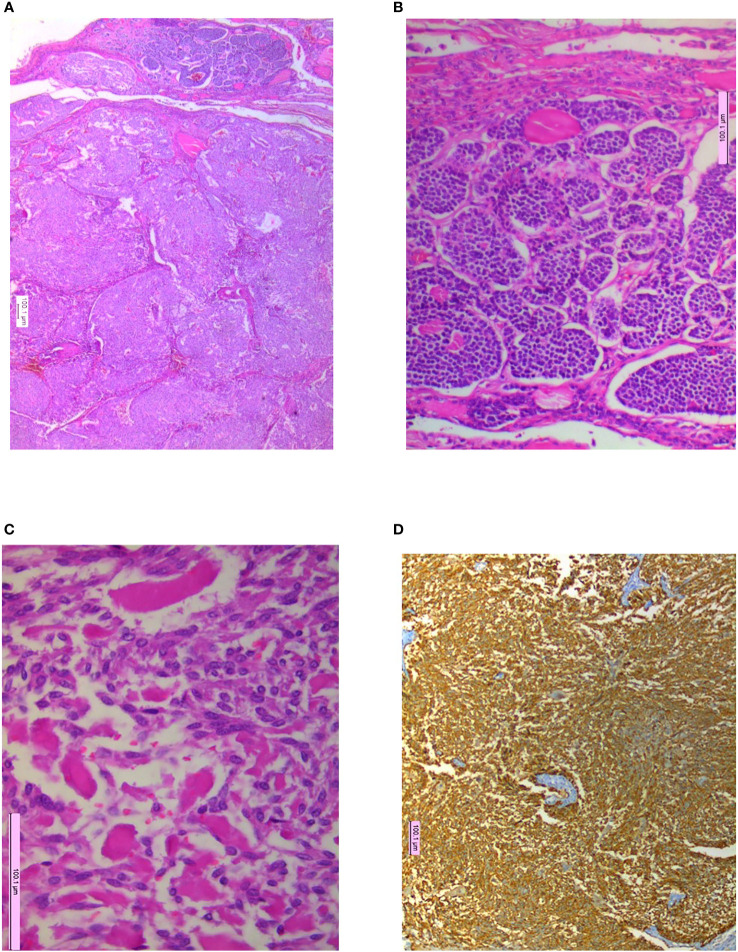
Microphotographs showing **(A)** nodules of tumor separated by fibrous septa with capsular invasion, H&E 10×; **(B)** organoid pattern of monomorphic cells, H&E 20×; **(C)** pale homogenous material interspersed within tumor cells, H&E 20×; **(D)** immunohistochemistry (IHC) for calcitonin with strong cytoplasmic positivity, IHCx.

**Figure 2:**
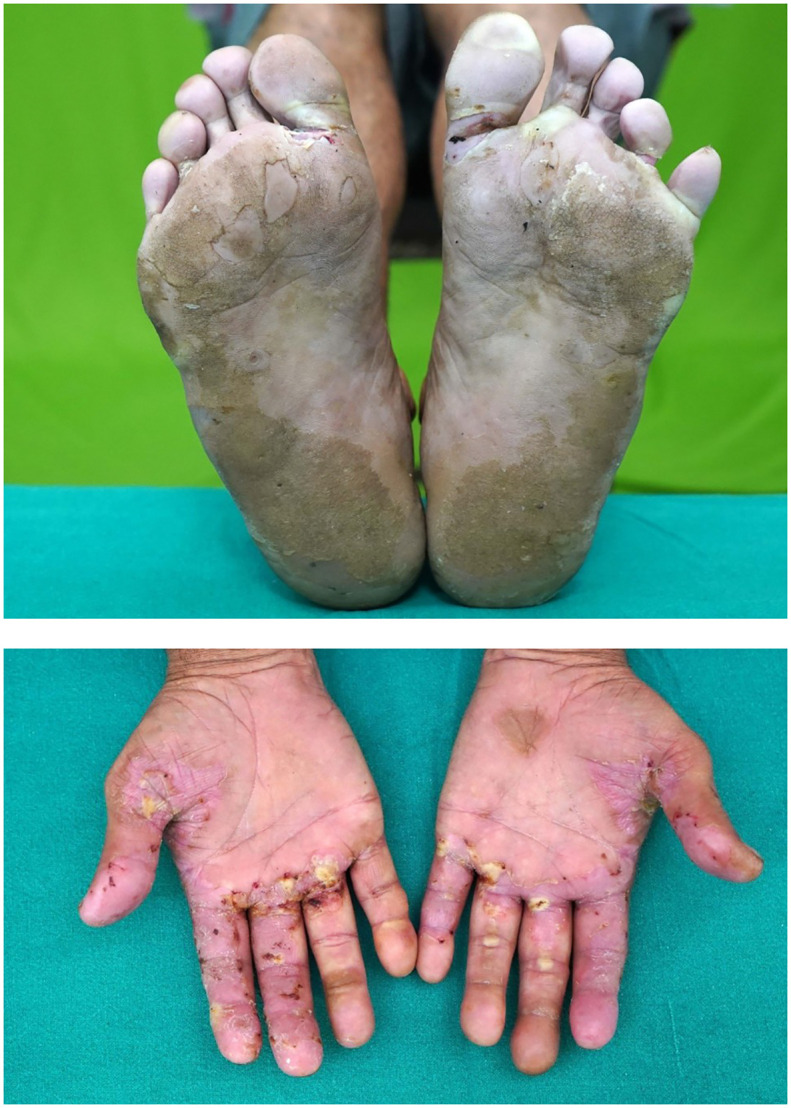
This 52-year-old male patient having stage IVB MTC was put on sorafenib 200 mg once a day and was shown to have hand–foot syndrome after 2 months of therapy. The lesions were gradually resolved following dose reduction.

**Figure 3:**
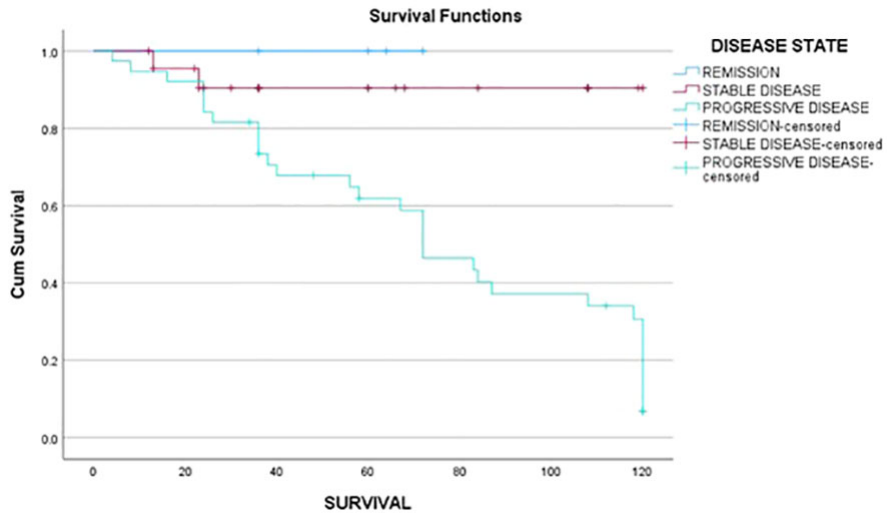
Progression-free survival of subjects in relation to stable disease and progressive disease at first follow-up.

**Figure 4:**
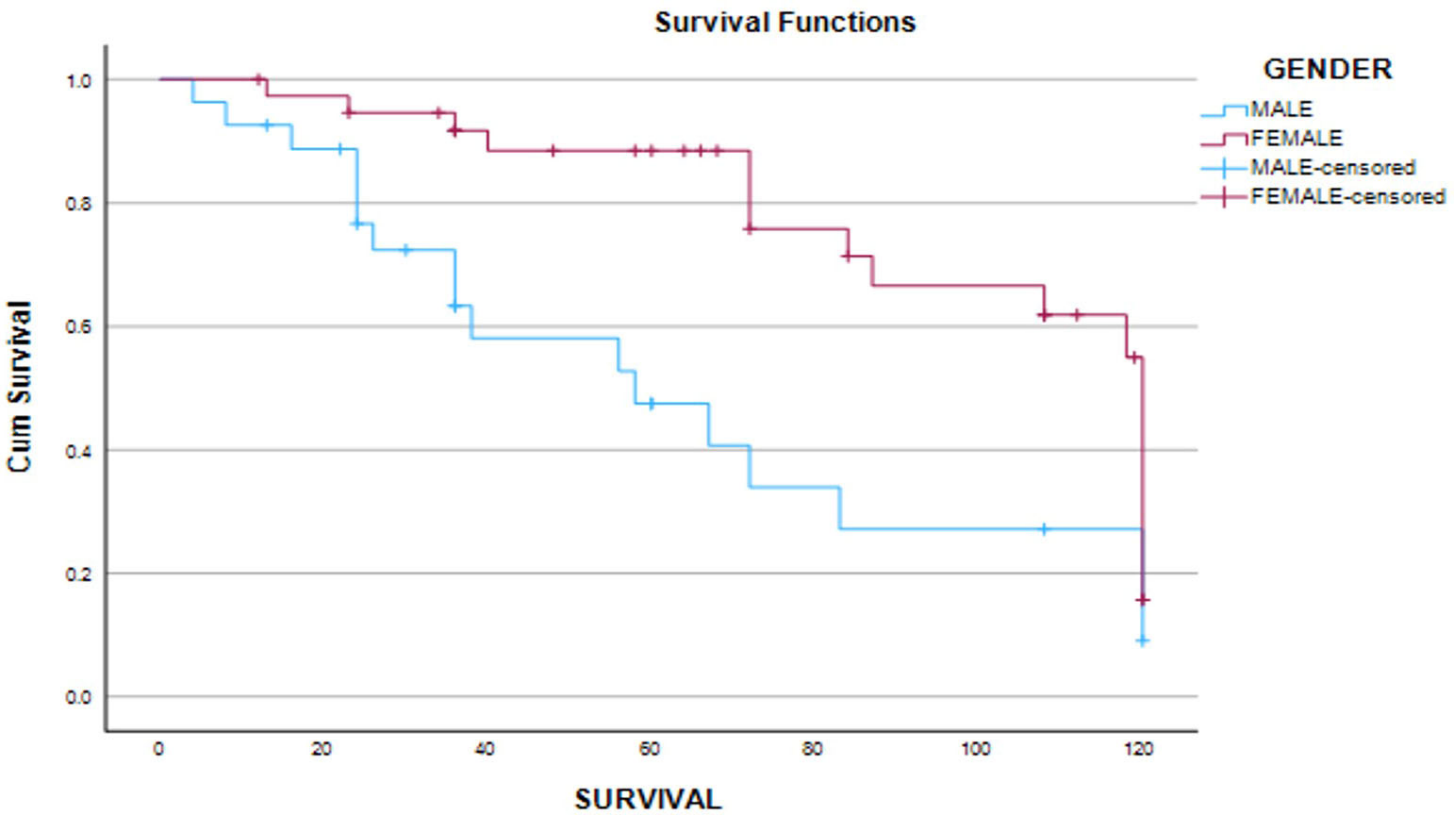
Progression-free survival of subjects in relation to gender.

Histopathological specimens available for analysis showed positivity for calcitonin (*n* = 64) and chromogranin (*n* = 36) as immune histochemistry hallmark, whereas capsular invasion was seen in 46 (59%) cases (**Figures 1**–**4**).

Surgery was the primary modality of therapy offered to 73 (93.6%) patients. Primary medical therapy and primary radiotherapy were offered to one and two patients, respectively, due to inoperable disease. Tumor surveillance was offered to one patient who had less than 2-cm lesions without lymphadenopathy. Eleven patients underwent initial lobectomy upon FNAC suggestion of suspicious but inconclusive results. They subsequently underwent a complete thyroidectomy after being proven to have MTC on histopathology. According to the available surgical report (*n* = 54), the most employed primary surgical strategy was total thyroidectomy with central node dissection (58%), followed by total thyroidectomy with modified radical dissection (31%). The most important surgical complication encountered was hypoparathyroidism, seen in 48 patients, all of whom were supplemented with calcium and active vitamin D. Hoarseness of voice arising from recurrent laryngeal nerve palsy was seen in 16 patients. One patient with extensive disease was put on a nasogastric tube for feeding for more than 4 months. The most common indication of modified radical neck dissection was stage of the disease.

Among available data (*n* = 73), 40 (54%) had progressive disease, 29 (40%) had stable disease, and 4 were in remission after the primary modality of therapy.

The median percent fall in calcitonin level after surgery at the first follow-up visit was 94% (IQR 81%–98%) (*n* = 37). The percent fall in calcitonin was dependent on the tumor stage at presentation (Mann–Whitney *U* 31.5; *p* = 0.001) with less fall in stage IV disease.

External beam radiotherapy was offered to 29 patients with a median dose of 30 Gy. Twenty-five patients received TKIs as second-line or third-line therapy; among them, 18 patients received sorafenib, 10 received lenvatinib, and 5 received cabozantinib. Five patients who received sorafenib therapy initially were shifted to lenvatinib on later visits. The most common reason for shifting sorafenib to lenvatinib was progressive disease, followed by adverse effects. Four patients were shifted to cabozantinib from lenvatinib on later visits for progressive and dedifferentiated disease.

Nine patients received PRRT with Lu-177 plus capecitabine therapy without any antecedent TKI therapy.

Among the *RET*-positive cases, nine patients received TKI viz sorafenib (six), lenvatinib (two), and cabozantinib (one). Four patients later shifted to cabozantinib once it was available mainly because of the progressive shortening of calcitonin doubling time and adverse reaction to sorafenib and lenvatinib.

**Figure 5:**
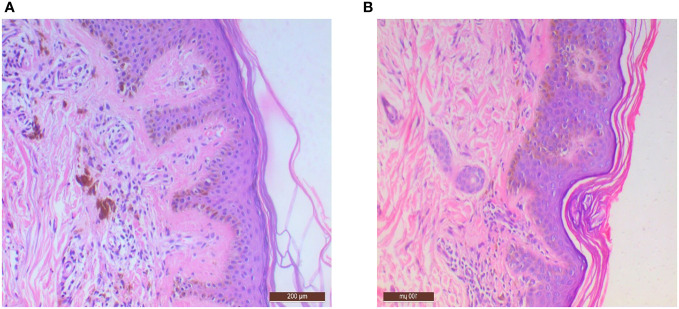
Microphotographs showing **(A)** mild acanthosis with pale eosinophilic material deposition in papillary dermis with melanin incontinence, H∓E 10×; **(B)** lichenoid deposition of amyloid with mild perivascular inflammation, H∓E 10×.

**Figure 6:**
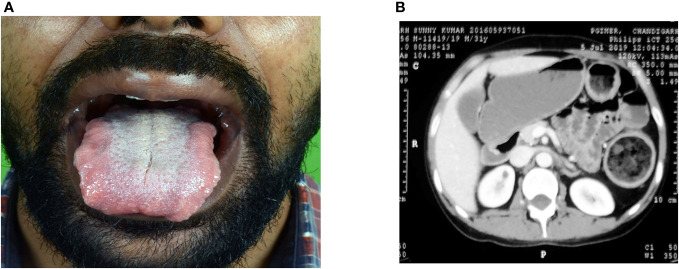
A 27-year-old male patient presented with MTC, mucosal neuroma, and bilateral pheochromocytoma with dilated gut loops, and diagnosed to have MEN. **(A)** Mucosal neuroma. **(B)** Dilated gut loops.

Seventeen patients had hand–foot syndrome in the form of desquamation of palm and sole, and one patient had MPGN as an adverse event of TKI therapy, which was resolved after stopping therapy (**Figures 5**, **6**).

Follow-up data were available for 64 patients. As per the last follow-up data, the median follow-up duration was 58 months (IQR 28–108 months). Twenty-four (33%) had progressive disease, 19 (26%) had stable disease, 11 (15%) were in remission, and 10 (14%) patients had succumbed to their illness. Fourteen patients were lost to follow-up. Among *RET* mutation-positive patients (*n* = 22), 8 had progressive disease, 7 had stable disease, 4 were in remission, and 3 succumbed to their illnesses. The median dose of sorafenib, lenvatinib, and cabozantinib used was 400 mg, 20 mg, and 120 mg, respectively.

The median progression-free survival (PFS) time was 60 months (95% CI 35–108). Patients having remission (62 months; IQR 28 months), stable disease, and no biochemical and structural residue after the primary modality of therapy (36 months; IQR 85 months) (Log-rank 10.4; *p* = 0.005) had a better PFS as compared to other patients. Female patients (Log-rank: 9.5; *p* = 0.002) had a better PFS (72 months; IQR 78 months) as compared to male patients (36 months; IQR 43 months). Overall survival was not dependent on gender, age at presentation, stage of disease, and post-primary therapy disease status.

Median overall survival and PFS were comparable in *RET*-positive and *RET-*negative patients. Thirty-seven patients (47.4%) had both biochemically and structurally persistent disease, 10 (12.8%) patients had only biochemically persistent disease, and 8 (10.3%) had only structurally persistent disease at the time of last follow-up.

## Discussion

In this ambispective study, 78 pathologically proven MTC patients have been dealt with. The overall age of presentation was the fourth decade and there was female preponderance. Female patients were presented earlier than male patients. The most common presenting symptom was goiter, and the median duration of the symptom was 23 months. Nearly two-thirds of the patients were diagnosed with FNAC among the patients subjected to it. Germline *RET* mutation was positive in 28% of patients; however, 60% of the patients were evaluated for *RET* mutation status. *RET* mutation-positive patients were presented at an earlier age (third decade) than others. Median PFS was better in patients with stable disease at first follow-up and in female patients. Median PFS was not related to *RET* mutation status. The addition of TKI following surgery with or without RT may prolong the survival of these patients. PRRT was also shown to be beneficial to some extent, but no conclusion can be drawn because the number of patients receiving PRRT was less.

### Epidemiology of MTC

The mean age of presentation (43 ± 11 years) was comparable with the global demographic data on MTC (3, 6). There was female preponderance (60%), and they presented at an earlier age (mean 42 years). A study from the Asian subcontinent showed a similar age of presentation (42.88 ± 2.67 years) but a different male-to-female ratio (2:1) as compared to the present study (7). Two previous studies from the southern part of India showed a similar mean age of onset in both genders (male, 41.7 years vs. female, 42.3 years); in contrast, the present study showed an earlier age of onset in female patients (8, 9). The median duration of the presentation was 23 months (IQR 12–35 months), which was lesser compared to previous studies (7, 8).

MTCs are mainly sporadic, comprising 75% to 80% of cases in different series. *RET* mutation is the most common among familial cases. Rare *RET* mutation-negative familial cases and ER-β-positive cases have also been reported (10, 11). Twenty-two patients (46%) had germline *RET* mutation-positive in this cohort. Though no gender difference was seen in *RET* mutation-positive MTCs in literature, the present study again showed a female preponderance (*n* = 13) (6–8, 12) and an early age of presentation in *RET*-positive patients. The most common *RET* mutation encountered was codon 634, which was similar to another Indian study (13).

### Clinical presentation of MTC

The most common presenting complaint was goiter with lymphadenopathy followed by weight loss and diarrhea. A review by Elisei et al. from Pisa, Italy concluded that most of the MTCs are diagnosed in a subject with a thyroid nodule, either single or in the context of multinodular goiter, without any other specific symptom; however, untreatable diarrhea and/or flushing can be present in very few mostly advanced cases (14). A study from India showed goiter with cervical lymphadenopathy was the most common presenting symptom seen in 67% of patients in their cohort (15). In another Southeast Asian study, 81.2% of the study patients presented with goiter. Metastasis was present in 25% of cases (7). Among the less frequently reported presentations, one patient had ectopic Cushing’s syndrome, one was detected during evaluation for prostatic malignancy, two had thyrotoxicosis, and one patient had hypertensive crisis presented during pregnancy due to pheochromocytoma.

Stage IV disease was the predominant stage at presentation (53%); more male patients had stage IV disease than female patients. This is possibly due to the fact that cosmetic issues of goitrous lesions lead to earlier detection. Predominant stage IV presentation in this study was similar to other studies (16, 17). Though sequential involvement of the regional lymph nodes, lungs, and bones is logical because of the lymphatic spread of the disease, it is not the rule as the prevalence of lymph node metastases at diagnosis varied between 25% and 62% in several other studies (18, 19).

### Laboratory surrogates and MTC

The median serum calcitonin level (*n* = 64) and CEA level (*n* = 39) at presentation was 1,274 pg/mL (IQR 370–4,923 pg/mL) and 149 ng/mL (IQR 30–316), respectively, which was in linear relation with the stage of disease at presentation as shown in other studies (8). In advanced and de-differentiated disease, CEA level surpassed calcitonin level (20). Although routine evaluation of solitary thyroid nodules with serum calcitonin level is not recommended by any of the society guidelines due to lack of uniform data, lack of cost-effectiveness, and resource constraints (21–23), serum calcitonin in the background of nodular thyroid diseases allows the preoperative diagnosis of unsuspected sporadic MTC as seen in this study. Levels of calcitonin can also predict the stage of the disease (24–27). Because of the high prevalence of nodular thyroid disease at our center, the initial evaluation protocol is USG of the neck followed by FNAC and then biochemical evaluation.

FNAC was done in 38 patients, 22 (58%) of whom were diagnosed to have MTC on cytopathology, 4 had differentiated thyroid carcinoma, and 5 had benign disease on initial reporting. FNAC was reported as 63% sensitive in detecting MTC (28).

### Treatment of MTC

Surgery was the primary modality of therapy offered to 94% of patients, which was similar to other studies. Non-resectability of the lesion resulted in primary RT and primary TKI in 5% of our study subjects. Tumor surveillance was offered to one patient, with a negligible disease burden. Eleven patients (15%) underwent initial lobectomy followed by completion thyroidectomy after being proven to have MTC on histopathology. At first follow-up, 54% had progressive disease, 40% had stable disease, and 5% were in remission. The median fall in calcitonin at the first follow-up visit was 94% (IQR 81%–98%) (*n* = 37). Radiotherapy in the form of external beam radiotherapy was offered to 29 patients with a median dose of 30 Gy. Twenty-five patients received TKIs as second-line or third-line therapy, which was concurrent with the current guideline (29). The most commonly used TKI was sorafenib followed by lenvatinib and cabozantinib. Majority of the time selection of primary TKI was based on the availability and affordability of the patients. As far as the adverse effect profile is concerned, all TKIs had similar adverse events like desquamation of hand and foot and oral mucosa, and gastrointestinal intolerance. The most common reason for shifting sorafenib to lenvatinib was progressive disease followed by adverse effects. Similarly, progressive disease was the main reason behind shifting patients from lenvatinib or sorafenib to cabozantinib. Although cabozantinib was available for the last 1 year in the author’s institute, it showed greater control over biochemical and/or structural disease progression as far as the last 1 year’s data are concerned. Treatment of a progressive disease with TKI showed stable disease only in 26% of patients in this study, which is far less than the global data that showed moderate therapeutic benefit with 73% of patients reported to have either stable disease or partial response as per a recent metanalysis (30). Nine patients received PRRT with Lu-177 in combination with capecitabine [data of eight patients have been published elsewhere (31)], of whom six had stable disease. Biochemical response with reduction of calcitonin level was observed in three patients and no therapy-limiting adverse effects have been observed. Another Indian study showed similar results with a 60% response rate in terms of efficacy and similar safety profile of the modality (32). Some of the Indian centers also tried metaiodobenzylguanidine (MIBG) as the diagnostic and therapeutic modality but detection and response rate were not satisfactory. Sensitivity of MIBG scan was 30%, and 30% of the study population had stable disease or partial response with MIBG as therapeutic modality (33).

### Follow-up of MTC

The median follow-up duration was 58 months. Twenty-four patients (32.9%) had progressive disease, 19 (26%) patients had stable disease, 11 patients (15%) were in remission, and 10 (13.7%) patients had succumbed to their illness. Fourteen patients were lost to follow-up. Among RET mutation-positive patients (*n* = 22), 8 had progressive disease, 7 had stable disease, and 4 were in remission. Three patients succumbed to their illnesses in the *RET* mutation-positive cohort. The median PFS time was 60 months (95% CI 35–108 months). Patients had stable disease and disease remission at first follow-up and female patients had a better PFS as compared to others.

### Strength of the study

Given the infrequency of MTC diagnosis and limited data, our study represents a commendable effort to comprehensively analyze data from histopathologically confirmed MTC cases particularly within the Indian context. The strength of the study is the largest cohort from north India and nearly 46% of patients were subjected to genetic testing. This study has the highest number of patients who received TKI among all studies from the Indian subcontinent.

### Limitation

The major limitations of the study are its ambispective nature, incomplete data, heterogeneity of presentation, and cases being handled by different surgeons, resulting in the lack of uniformity in surgical approach. A long study period and the change in the standard of care might have affected the therapeutic decision-making in many of those cases. MEN-related information, viz., biochemical parameters in some of the cases, was limited due to the unavailability of detailed records.

## Conclusion

MTC comprises 3% of thyroid malignancies presented in this cohort with a female preponderance. Germline *RET* mutation-positive patients show an early age at presentation. Surgery was the first-line therapy followed by radiotherapy and TKI. The median PFS of this cohort was comparable with global data and female gender and disease remission or at least stable disease at first follow-up had a better PFS as compared to others.

## Data availability statement

The raw data supporting the conclusions of this article will be made available by the authors, without undue reservation.

## Ethics statement

The studies involving humans were approved by Institute Ethics Committee of PGIMER. The studies were conducted in accordance with the local legislation and institutional requirements. The participants provided their written informed consent to participate in this study. Written informed consent was obtained from the individual(s) for the publication of any potentially identifiable images or data included in this article.

## Author contributions

AC and AR: overall data collection and compilation and manuscript writing. DD and NP: Surgical section analysis and formatting of text. US: Histopathological analysis. RP and SM: Manuscript editing and statistics. PD and SB: Over all conceptualizations and manuscript editing. PD: Correspondence and over all idea. All authors contributed to the article and approved the submitted version.
